# Effects of a novel combination of orlistat and acarbose on tolerability, appetite, and glucose metabolism in persons with obesity

**DOI:** 10.1002/osp4.405

**Published:** 2020-02-07

**Authors:** Ulf Holmbäck, Anders Forslund, Stefan Grudén, Göran Alderborn, Arvid Söderhäll, Per M. Hellström, Hans Lennernäs

**Affiliations:** ^1^ Department of Public Health and Caring Sciences, Clinical Nutrition and Metabolism Uppsala University Uppsala Sweden; ^2^ Department of Women's and Children's Health Uppsala University Uppsala Sweden; ^3^ Empros Pharma AB Solna Sweden; ^4^ Department of Pharmacy Uppsala University Uppsala Sweden; ^5^ Department of Medical Sciences Uppsala University Uppsala Sweden

**Keywords:** gastrointestinal tolerability, modified release, obesity, type‐2 diabetes

## Abstract

**Objective:**

There is an unmet medical need for a safe and effective weight loss product with minimal systemic side‐effects. In this study, the effect of a novel modified‐release fixed‐dose combination of orlistat and acarbose (MR‐OA) was compared with conventional orlistat (CO) regarding tolerability, appetite and glucose metabolism.

**Methods:**

Sixty‐seven men with obesity, aged 24 to 60 years with body mass indexes (BMIs) 33 to 40 kg m^−2^ or BMIs 30 to 32 kg m^−2^ and waist circumference above 102 cm were included. They were randomized to either three different doses of the test formulation MR‐OA (60 mg orlistat/20 mg acarbose, 90/30 and 120/40) or CO (Xenical, 120 mg orlistat) for a 2‐week study of daily treatment. The participants spent days 1 and 14 at the clinical research centre where they received standardized meals, had blood sampling and filled in questionnaires regarding tolerability and appetite after meals. In days 2 to 13, the participants were at home and continued to fill in the questionnaires daily.

**Results:**

In the MR‐OA groups, reports of liquid and oily stools as well as faecal incontinence were fewer, whereas reports of gastric distension and flatulence were higher, compared with the CO group. More participants reported decreased hunger in the 90/30 and 120/40 MR‐OA, and postprandial plasma glucose concentration was reduced in all MR‐OA groups compared with CO.

**Conclusions:**

This study shows that by using a modified‐release dosage form, orlistat and acarbose can be combined without compromising tolerability. Furthermore, MR‐OA shows promising effects regarding reduction of appetite and reduces postprandial glucose. Tolerability is coupled to compliance and thereby efficacy of a treatment; therefore, this novel combination MR‐OA could be an effective approach for weight loss treatment. A follow‐up study in a more diverse population and for a longer duration with weight loss as primary outcome variable is planned.

**Abbreviations**
CRC: clinical research centre
GI: gastrointestinal
GLP‐1: glucagon like peptide‐1
LOCF: last observation carried forward
MR‐OA: multiple release orlistat acarbose
TID: three times a day
VAS: visual analogue scale

## 1 INTRODUCTION

With the exception of orlistat in its conventional oral form, all currently approved, anti‐obesity pharmaceutical products and most products currently in development enter the bloodstream, which can lead to unwanted systemic effects.^1^, ^2^, ^3^ Of all patients eligible for obesity pharmacotherapy, few are prescribed available medications and even fewer use them.^4^, ^5^ The reasons for this lack of use might be related to costs as well as safety concerns or side‐effects.^5^ Orlistat has been used for decades with no serious side‐effects.^6^ It reversibly inhibits dietary lipid digestion in the gastrointestinal (GI) lumen, which leads to a modest weight loss. Orlistat's efficacy, as well as attrition, is similar to the other weight loss products^7^; however, orlistat treatment in the conventional oral dosage forms is associated with frequent (~20% of patients) GI side‐effects, such as liquid and oily stools.^8^ These GI‐side‐effects have an impact on compliance.^10^ and after 1 year of treatment, about 30 to 50% of the patients had stopped taking the medication. 11, 12 Moreover, a particularly unfortunate side‐effect of conventional orlistat is that it stimulates appetite and feelings of hunger.^13^, ^14^, ^15^, ^16^, ^17^ Therefore, there is an unmet medical need for a safe and effective weight loss product with minimal systemic side‐effects and preferably with less GI side‐effects.

In an attempt to develop an improved formulation with orlistat's effects on weight loss product, albeit with less GI side‐effects, a combination of orlistat with acarbose was tested. Acarbose (Glucobay), like orlistat, only acts locally in the GI tract.^18^ Acarbose delays carbohydrate digestion in the GI tract and reduces the subsequent intestinal glucose absorption rate.^19^, ^20^, ^21^ On its own, acarbose has also been shown to have a small effect on weight loss^22^, ^23^, ^24^; however, acarbose treatment in its conventional oral dosage forms is associated with frequent (~3‐30% of patients) GI side‐effects, mainly flatulence and sometimes soft stools or abdominal discomfort.^25^, ^26^

In this exploratory study, an oral modified‐release (MR) multiple unit, based on a fixed‐dose combination of orlistat and acarbose (MR‐OA), was investigated. This formulation includes distinct release rates of each drug in different GI compartments, with the aim of reducing untoward GI side‐effects. Moreover, by changing lipid and carbohydrates digestion, the aim was to trigger neuroendocrine feedback signalling systems and thereby affect appetite. In addition, as glucose metabolism usually is affected in people with obesity and the risk of developing diabetes type 2 is increased,^27^ this combination hade the additional benefit of the effect of acarbose on the postprandial glucose response.

## 2 MATERIAL AND METHODS

### 2.1 Study design

This clinical trial was conducted as a single‐centre, controlled, randomized, parallel‐group, phase IIa pilot study with 2 weeks of daily treatment (three times a day [TID]) during meals and three different doses of the test formulation orlistat/acarbose (MR‐OA), namely, 60/20, 90/30 and 120/40 mg mg^−1^. The results were compared with conventional orlistat (Xenical, 120 mg). Glucobay (conventional acarbose) was not chosen as a comparison at this stage, as Glucobay on its own is not a weight‐loss medication and a combination of conventional orlistat and conventional acarbose is not recommended.^28^ Two weeks were chosen as the study length to allow the participants to acclimatize to the acarbose component in the MR‐OA.^29^ The trial was registered at EudraCT (2016‐001055‐50), where a clinical study report is filed.^30^ The clinical trial was performed by the contract research organization Clinical Trial Consultants in Uppsala, Sweden. The primary objective of the trial was to compare the appetite/tolerability score of the test formulation (EMP16‐01 90/30) with the reference product (Xenical). The appetite/tolerability score was calculated as the ratio between subjective appetite score (sum of appetite questions, measured with questionnaire) and GI symptoms score (sum of GI symptoms such as diarrheal, flatulence, oily spotting, gastric distention and frequency and intensity of nausea and pain, measured with questionnaire). Ethics approval was granted by the regional Ethical Review Board in Uppsala, Sweden (dnr 2016/257). All study participants signed the consent form.

### 2.2 Study participants

**Figure 1:**
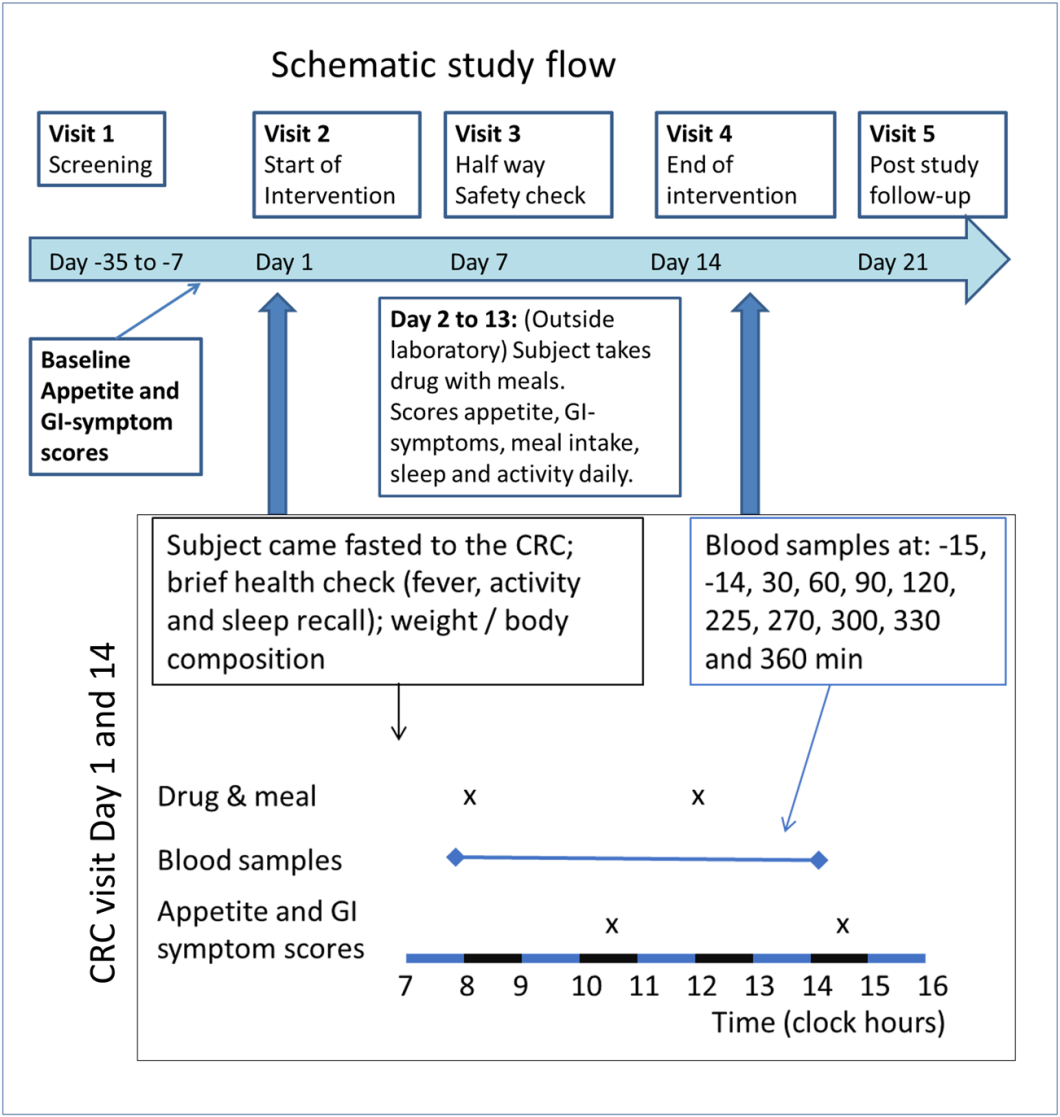
Visits and procedures during the study

Male participants 24 to 60 years of age with either body mass index (BMI) 33 to 40 kg m^−2^ or participants with BMI 30 to 32 kg m^−2^ and a waist circumference above 102 cm were included. A person with greater muscle mass also can have a BMI of around 30 kg m^−2^, but such persons would not be a target group for the study drug. Therefore, a waist circumference criterion was added to the inclusion criteria to ensure the enrolment of a relevant population.^31^ Women were not included at this stage, mainly because the menstrual cycle influences glucose metabolism^32^ and appetite,^33^, ^34^ which would increase variability in this 2‐week pilot study. Furthermore, women and men differ in various components relating to eating behaviour and responses to food cues.^35^ To avoid residual endocrine effects of late puberty, adults between 18 and 24 years were excluded. Participants were screened for eligibility as per study‐specific inclusion/exclusion criteria in the 5 weeks prior (days −35 to −4) to randomization and the first oral administration of the test and reference products (Figure 1). Additional inclusion criteria were based on medical history, physical findings, vital signs, ECG and laboratory values at the time of screening.^30^ All participants had an adequate glucose control (none had a previous diagnosis of diabetes mellitus type II); serum creatinine levels <1.5 times the upper limit of normal, serum ASAT, ALAT, ALP and GGT levels < 2.5 times upper limit of normal; and serum bilirubin levels < 1.5 times upper limit of normal.

### 2.3 Treatments

Eligible and consenting participants arrived at the clinical research centre (CRC) in a fasting condition (at least 8 h since last meal) on the morning of the first study day (visit 2, day 1) and were randomly assigned to one of four parallel treatment groups (see below). The study compared four test products: 60/20, orlistat (60 mg) plus acarbose (20 mg); 90/30, orlistat (90 mg) plus acarbose (30 mg); 120/40, orlistat (120 mg) plus acarbose (40 mg); and conventional orlistat (120 mg; Xenical) was used as a reference. One of the three dosages of the test product (MR‐OA) or the reference drug product (conventional orlistat) was taken orally together with the three main daily meals (fed state). Each subject was instructed to take the study product half‐way through the meal together with 50 mL of water.

### 2.4 Study outline

This clinical trial consisted of five visits to the clinic, including screening and follow‐up (Figure 1). The total duration of the treatment with the study drug products was 2 weeks for each volunteer with no overnight stays. Visit 1 included screening and health investigation and occurred 1 to 5 weeks prior to study start. On days −1 through −3, baseline questionnaires were completed by each subject outside the clinic. On study days 1 and 14, each subject received two meals during the visit (Figure 1). Meal items were based on Swedish dietary habits but with a somewhat elevated fat content to ensure sufficient amounts of dietary fat to test the effect of orlistat. Breakfast was rye bread with butter, cheese and salami (846 kcal, 16 energy percent [E%] protein, 50 E% fat and 32 E% carbohydrates). Lunch was ham and cheese pie and white bread with butter (1,053 kcal, 14 E% protein, 55 E% fat and 31 E% carbohydrates). The clinic visit ended at 1,600, and during dinner as well as during the remaining days, the drug product was self‐administered TID by each subject, together with all three daily main meals. Throughout the trial, the participants recorded appetite (eg, hunger and desire to eat), GI symptoms, sleep and physical activity approximately 2 h after each main daily meal using a questionnaire (see below for description). There were no dietary restrictions during the study days at home except that participants had to abstain from alcohol for 24 h and from food and drink for at least 8 h prior to visits 1, 2 and 4. Visit 3, on day 7 of the experimental period, included a safety check‐up in which plasma/serum concentrations of liver enzymes were assessed. At visit 5, about one week (4‐10 d) after end of the experimental period, another safety follow‐up was performed. On day 21 (visit 5), a final follow‐up visit was performed at the clinic.

### 2.5 Efficacy variables

GI symptoms were assessed during the 3 d before randomization (baseline) and during the whole 14‐d period using the Gastrointestinal Symptom Score (GSS). The GSS was a slightly modified version of a previous orlistat‐only‐based questionnaire,^36^ which additionally contained the most common GI side‐effects of acarbose (Table S1).^37^ The first part of the GSS (questions 1‐7) investigated quantitative side‐effects and the second part (questions 8‐13) subjective side‐effects. Using the same principal as Cavaliere et al (2001),^36^ side‐effect scores were not linear with frequency of events (Table S1). Some items were clarified, and the scoring employed by Cavaliere et al (2001)^36^ was adjusted with the aim to better reflect the patients' perceptions of the side‐effects (Table S1). The GSS questionnaire was written in Swedish (all researchers and study participants were fluent readers). The wording of the questions was based on the Swedish patient information brochures for Xenical and Glucobay.^28^, ^38^

Subjective appetite was assessed during the 3 d before randomization (baseline) and during the whole 14‐d period. Participants were asked to rate sensations of hunger, fullness, prospective food consumption and desire to eat.^39^ Answers were entered on a 10‐point Likert scale with verbal anchors at the end of the scales.^39^ Change in appetite score relative to baseline was calculated for the total 14 periods. Also, the number of participants with a clinically relevant change in daily hunger scores (see Section 2.6) was calculated.

The participant's meal patterns were assessed during two 3‐d periods, ie, the 3 d preceding days 1 and 14, using a short diary.^40^ Meal patterns were categorized as “Good,” defined as having breakfast, lunch and dinner each of the 3 d, ie, nine main meals during the three‐day period; “Okay” (six to eight main meals during the 3‐d period) and “Poor” (≤5 main meals during the 3‐d period). These definitions were based on tentative findings of a relationship between meal intake and diet quality,^41^ and fewer meals in general indicate that meals have been replaced by snacking (eating food of lower diet quality), but not a reduction of energy intake.

Glucose, insulin and GLP‐1 were repeatedly assessed at visits 2 and 4. Blood was drawn from a peripheral vein using a stationary catheter. Glucose and insulin were collected in ethylenediaminetetraacetic acid‐tubes and analysed at the Uppsala University Hospital laboratory. Glucose was analysed using Abbott Architect (Abbott Diagnostics, Lake Forest, Ill). Insulin was analysed using immunoassays (Cobas E602, Roche Diagnostics, Indianapolis, IN, USA). Blood for analyses of glucagon like peptide‐1 (GLP‐1) was collected in P800‐tubes. After centrifugation, the supernatant was transferred to polypropylene tubes and stored at −70 °C before analysis. GLP‐1 was analysed by Mercodia AB (Uppsala, Sweden) using Mercodia total GLP‐1 NL‐ELISA (CAT# 10‐1278‐01). The enzyme‐linked immunosorbent assay (ELISA) and radioimmuno‐method were validated for intended use, including precision and accuracy, selectivity, range of quantification and sample stability. The precision of the GLP‐1 analysis was a total coefficient of variation (CV) of 9.2% for quality control (QC) low, 5.6% for QC medium and 4.4% for QC high with a LLOQ of 1.0 pmol L^−1^. Maximum achieved concentration *C*_max_ and mean concentration during blood draws, *C*_mean_, was calculated for days 1 and 14 of the 14‐d experimental period.

### 2.6 Statistical analysis

A statistical analysis plan was developed ahead of study start together with the CRC. No previous research has defined a clinically relevant change in tolerability. Therefore, power calculation was based on change of appetite: With a sample size of 12, a difference in hunger of about 10% would be detected.^42^ This would enable the detection of a difference of 1.5 points somewhat equivalent to a ≥15‐ to 25‐mm difference when using a 100‐mm visual analogue scale,^43^ which has previously been shown to elicit a change in energy intake.^44^ The inclusion goal was set at 15 participants to allow for drop‐outs. A change over two categories (eg, from mild to severe), equivalent of a change of ≥4 points in GSS, was defined as a clinically relevant difference. Power and sample size were calculated with G*Power version 3.1.9.2 (University of Cologne, Cologne, Germany). The Intention‐To‐Treat (ITT) data set was used with a last observation carried forward (LOCF) approach. For questionnaires, at least seven measurements from a specific patient were needed for the LOCF. When the denominator was zero when calculating ratios, either the LOCF approach or median for that particular group was used. Differences between the three MR‐OA groups (60/20, 90/30 and 120/40) and the reference product group (conventional orlistat, Xenical) were tested with Welch's *T* test for continuous data and Wilcoxon rank sum test for ordinal data. Data are presented as mean ± SD/SEM or median ± semi‐interquartile range. Significance was set at α= 0.05. Adjustments for multiple comparisons were made according to Holm.^45^ Statistical analyses were performed with R Commander version 2.4‐2.^46^

## 3 RESULTS

**Figure 2:**
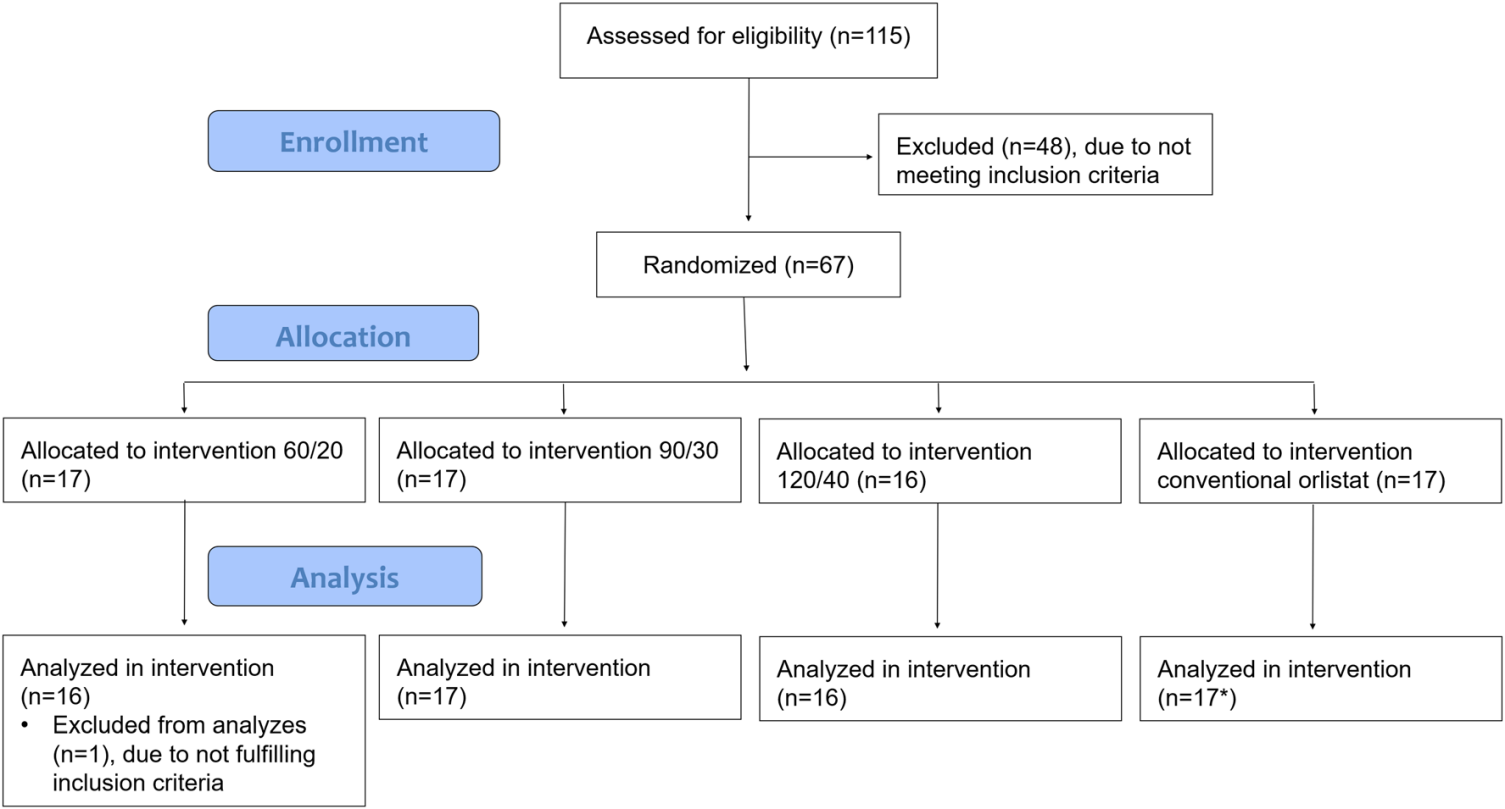
Patient flow in the clinical phase IIa study with three different doses of the test product multiple release orlistat acarbose (60/20, 90/30 and 120/40; orlistat/acarbose respectively) and one dose of conventional orlistat (120 mg). *Two drop‐outs during the 14‐d experimental period

In this study, 115 men with obesity were screened, 67 of whom were included and 64 of whom completed the trial (Figure 2). Due to a mix‐up of blood samples, seven additional participants had to be randomized. One subject under the inclusion age was mistakenly randomized in the 60/20 group (not included in the ITT set), and there were two drop‐outs in the conventional orlistat group (included in the ITT set). Baseline characteristics are displayed in Table 1. There were no baseline differences in these variables between any groups.

**Table 1:** Baseline characteristics of the four treatment arms (mean ± SD)

Treatment	Age, Years	Weight, kg	BMI, kg m^−2^	Body Fat, %	Waist Circumference, cm
60/20	40.5 ± 8.7	112.9 ± 16.2	34.5 ± 3.0	32.7 ± 2.7	119 ± 9
90/30	43.4 ± 8.5	110.4 ± 12.9	34.1 ± 2.8	31.8 ± 4.7	119 ± 8
120/40	42.3 ± 9.6	112.3 ± 9.9	34.8 ± 2.3	32.2 ± 4.6	121 ± 10
Conventional orlistat	45.5 ± 9.8	112.3 ± 12.9	35.4 ± 2.8	32.8 ± 4.9	119 ± 9

There were no differences in total 14‐day GSS (gastric symptom score) between the MR‐OA groups and conventional orlistat (Table 2). For several of the individual questions, lower scores were reported in the MR‐OA groups compared with conventional orlistat concerning the more orlistat‐related side‐effects (liquid and oily stools; Table 2). For some of the more acarbose‐related side‐effects (such as flatulence and gastric distension), higher ratings were recorded in the MR‐OA groups compared with conventional orlistat (Table 2). The same pattern was seen when participants with a minimal clinically relevant change (at least four‐point change from baseline in GSS score) were tabulated. Fewer participants reported liquid and oily stools or faecal incontinence in the MR‐OA groups compared with the conventional orlistat group (Table 3). More participants reported gastric distension and flatulence in the MR‐OA groups compared with the conventional orlistat group (Table 3). In the 120/40 MR‐OA group, more participants reported flatulence with discharge, mild headache and mild nausea (Table 3).

**Table 2:** Change from baseline (data collected during 3 d preceding the 14‐d period) in total gastric symptom score (GSS) and individual tolerability questions during the whole 14‐d period (median ± semi‐interquartile range)

Item	60/20	90/30	120/40	Conv. orlistat
Total gastric symptom score (GSS)	104 ± 39	114 ± 54	197 ± 70	144 ± 24
(1) How often have you been to the toilet for defecation?	7 ± 6	10 ± 4	25 ± 13	15 ± 12
(2) Have you had oily stools?	11 ± 7*	12 ± 8*	22 ± 9	28 ± 7
(3) How often have you had liquid stools?	5 ± 6*	6 ± 5*	20 ± 9	27 ± 10
(4) How often have you had flatulence with discharge?	13 ± 9	4 ± 10	15 ± 12	15 ± 7
(5) How often have you had oily spotting?	0 ± 1*	2 ± 4	8 ± 7	6 ± 7
(6) How often have you had faecal urgency?	5 ± 6	4 ± 4	8 ± 5	3 ± 2
(7) How often have you had faecal incontinence?	0 ± 0	0 ± 0	0 ± 1	0 ± 1
(8) Have you experienced nausea?	0 ± 0	0 ± 2	2 ± 2*	0 ± 0
(9) Have you experienced rectal pain?	0 ± 0	0 ± 0	1 ± 1	0 ± 0
(10) Have you experienced headache?	3 ± 8	2 ± 5	7 ± 9	0 ± 4
(11) Have you experienced gastric distention?	12 ± 8	2 ± 24	19 ± 17	8 ± 8
(12) Have you experienced gastrointestinal pain/discomfort	4 ± 3	1 ± 3	6 ± 9	4 ± 4
(13) Have you experienced flatulence?	37 ± 11	31 ± 24	47 ± 18*	19 ± 12

* *P*<.05 for being different from conventional orlistat (adjusted for multiple comparisons).

**Table 3:** Number of participants with a clinically relevant change (≥4 points compared with baseline) in GSS score during the whole 14‐d period in the four treatment arms (median ± semi‐interquartile range)

GSS Item	60/20	90/30	120/40	Conv. orlistat
(1) How often have you been to the toilet for defecation?	0 ± 0*	0 ± 1*	2 ± 1	2 ± 1
(2) Have you had oily stools? [≥3 points compared with baseline]	2 ± 1*	2 ± 2*	5 ± 1	5 ± 2
(3) How often have you had liquid stools?	1 ± 1*	0 ± 1*	2 ± 1	3 ± 1
(4) How often have you had flatulence with discharge?	0 ± 1*	0 ± 0*	3 ± 1*	1 ± 1
(5) How often have you had oily spotting?	0 ± 0*	0 ± 0*	1 ± 1*	2 ± 1
(6) How often have you had faecal urgency?	0 ± 0*	0 ± 0*	1 ± 0	1 ± 0
(7) How often have you had faecal incontinence?	0 ± 0*	0 ± 0*	0 ± 0*	1 ± 0
(8) Have you experienced nausea?	0 ± 0	0 ± 0	0 ± 0	0 ± 0
(9) Have you experienced rectal pain?	0 ± 0	0 ± 0	0 ± 0	0 ± 0
(10) Have you experienced headache?	2 ± 1	1 ± 0	3 ± 1*	2 ± 1
(11) Have you experienced gastric distention?	1 ± 1	3 ± 1*	3 ± 1*	0 ± 1
(12) Have you experienced gastrointestinal pain/discomfort	1 ± 1	0 ± 1*	2 ± 1	1 ± 1
(13) Have you experienced flatulence?	4 ± 1	6 ± 1*	7 ± 1*	2 ± 1

Abbreviation: GSS: gastric symptom score.

* *P*<.05 for being different from conventional orlistat (adjusted for multiple comparisons).

There were no differences in total rating score for hunger, fullness and desire to eat for the total 14‐d period between groups (Table 4). More participants in the MR‐OA 90/30 and 120/40 groups reported reduced hunger compared with the conventional orlistat group, using the stipulated 1.5 points difference in hunger rating per day as a cut‐off,^44^ after lunch and dinner, but not breakfast (Table 5).

**Table 4:** Change from baseline (data collected for 3 d preceding the 14‐d period) in ratings of hunger, fullness and desire to eat in the four treatment arms

Treatment	Hunger	Fullness	Desire to Eat
60/20	12 (−7‐31)	3 (−12‐33)	−7 (‐31‐1)
90/30	−2 (−15‐9	20 (−18‐58)	−8 (−21‐7)
120/40	−20 (−50‐31)	20 (−5‐55)	−26 (−64‐22)
Xenical	4 (−9‐27)	16 (−5‐34)	−6 (‐24‐11)

*Note.* Presented as median (interquartile range) of total 14‐d score. Questionnaire is presented in the supporting information.

**Table 5:** Number of participants with a 1.5 points/d decrease in hunger compared with baseline (data collected for 3 d preceding the 14‐d period) after breakfast, lunch and dinner in the four treatment arms

Treatment	After Breakfast	After Lunch	After Dinner
60/20	4 (2‐5)	1 (0‐1)*	2 (2‐3)*
90/30	4 (2‐4)	4 (4‐5)*	3 (3‐4)*
120/40	4 (2‐4)	5 (4‐5)*	5 (4‐6)*
Xenical	3 (2‐3)	3 (2‐3)	0 (0‐1)

*Note.* Presented as median (interquartile range) number of participants during the whole 14‐d period.

* *P*<.05 for being different from conventional orlistat (adjusted for multiple comparisons).

In the conventional orlistat group, a minority had good meal patterns before the study, whereas a majority in MR‐OA groups had good meal patterns. At the end of the study, participants in the conventional orlistat group had improved their meal patterns (no participants with poor meal pattern) and there were no differences between the groups (Table 6).

**Table 6:** Meal pattern before and at the end of the 14‐d phase IIa‐study in the four treatment arms

	Treatment Groups	Poor Meal Pattern	OK Meal Pattern	Good Meal Pattern	ALL
Pre‐dose	MR‐OA 60/20	1^a^	3	13	17
	MR‐OA 90/30	0	5	12	17
	MR‐OA 120/40	2	4	10	16
	Conventional orlistat	3	6	7	16
					
End of study	MR‐OA 60/20	0	2	13	15
	MR‐OA 90/30	0	2	15	17
	MR‐OA 120/40	0	5	11	16
	Conventional orlistat	0	2	12	14

*Note.* The data are presented as number of participants.

a Number of participants.

The plasma *C*_max_ of glucose was lower for all three MR‐OA groups during days 1 and 14 compared with conventional orlistat (Table 7). The postprandial plasma *C*_max_ of glucose was about 1 mmol L^−1^ lower at both days 1 and 14 after treatment with MR‐OA compared with conventional orlistat (Table 7). All three MR‐OA groups had lower *C*_mean_ glucose values than conventional orlistat at day 1 but not at day 14 (Table 7). The plasma *C*_max_ of insulin was lower during day 1, but not at day 14, for all three MR‐OA groups compared with conventional orlistat (Table 7). No differences were observed in insulin *C*_mean_ during day 1 or day 14. No differences in *C*_max_ or *C*_mean_ were observed for GLP‐1 between the groups (Table 7).

**Table 7:** Postprandial plasma concentrations of glucose, insulin and GLP‐1 (mean ± SD) at days 1 and 14, respectively, during the 14‐d study in the four treatment arms

Analyte	60/20	90/30	120/40	Xenical
Glucose *C*_mean_ day 1 (mmol L^−1^)	5.52 ± 0.57*	5.56 ± 0.58*	5.46 ± 0.51*	6.14 ± 0.72
Glucose *C*_mean_ day 14 (mmol L^−1^)	5.52 ± 0.61	5.66 ± 0.65	5.51 ± 0.59	5.96 ± 0.92
Glucose *C*_max_ day 1 (mmol L^−1^)	6.24 ± 0.64^*^	6.45 ± 0.75*	6.08 ± 0.66*	7.55 ± 1.17
Glucose *C*_max_ day 14 (mmol L^−1^)	6.32 ± 0.58^*^	6.48 ± 0.85*	6.16 ± 0.88*	7.49 ± 1.54
Insulin *C*_mean_ day 1 (pmol L^−1^)	33.51 ± 11.80	31.97 ± 19.74	30.91 ± 17.15	55.72 ± 42.24
Insulin *C*_mean_ day 14 (pmol L^−1^)	33.40 ± 10.40	33.52 ± 18.88	30.15 ± 16.23	47.22 ± 39.74
Insulin *C*_max_ day 1 (pmol L^−1^)	60.55 ± 19.76*	63.43 ± 34.44*	58.49 ± 33.02*	125.21 ± 93.31
Insulin *C*_max_ day 14 (pmol L^−1^)	67.81 ± 18.56	61.18 ± 27.80	61.34 ± 30.32	104.28 ± 84.30
GLP‐1 *C*_mean_ day 1 (pmol L^−1^)	14.25 ± 3.53	12.98 ± 2.98	14.97 ± 4.92	14.08 ± 4.31
GLP‐1 *C*_mean_ day 14 (pmol L^−1^)	15.69 ± 4.34	13.74 ± 2.37	15.25 ± 3.79	14.21 ± 3.82
GLP‐1 *C*_max_ day 1 (pmol L^−1^)	18.40 ± 4.69	18.10 ± 6.69	19.60 ± 6.82	17.44 ± 6.12
GLP‐1 *C*_max_ day 14 (pmol L^−1^)	23.29 ± 7.36	18.98 ± 4.88	21.21 ± 6.70	18.75 ± 4.91

Abbreviation: *C*_max_: highest concentration reached during blood sampling; *C*_mean_: average blood concentration from 0 to 360 min; GLP‐1: glucagon like peptide‐1.

* *P*<.05 for being different from conventional orlistat (adjusted for multiple comparisons).

Minor reductions were observed in body weight during the 14‐d study with no significant differences between the groups: MR‐OA 60/20, 90/30 and 120/40 and conventional orlistat lost (mean ± SD) 0.65 ± 0.93 kg, 0.59kg ± 1.00 kg, 1.31 ± 1.58 kg and 1.29 ± 1.53 kg, respectively. There were no changes in body composition or waist circumference (data not shown) in any of the four treatment groups.

## 4 DISCUSSION

This phase IIa trial in men with obesity showed that it is possible to combine OA without increasing GI side‐effects by using a MR formulation. If anything, the side‐effects were actually lower in this combination product compared with conventional orlistat. The multiple unit MR formulation of MR‐OA resulted in an improved GI tolerability as determined by the GI symptom score. Moreover, decreased hunger and postprandial glucose concentration were observed in the MR‐OA groups. As stated before, improving tolerability is a key component to increasing compliance,^10^ and thereby efficacy on a group level.

The MR‐OA groups reported less orlistat related side‐effects and more acarbose related side‐effects. The latter observation may appear obvious, as the conventional orlistat‐group did not receive any acarbose. Probably, the most “socially inconvenient side‐effect” of orlistat is faecal incontinence. Only one participant had one episode in each of the MR‐OA groups 60/20 and 90/30, and in the 120/40 group, six participants had one episode. In the conventional orlistat group, three participants had six or more episodes, another participant reported one episode and one reported two episodes. The conventional orlistat group reported liquid and oily stools more frequently. The most frequently reported acarbose‐related GI side‐effect was flatulence. It is important to note that no dose titration was performed in this study and that the participants in the MR‐OA 120/40 received 120 mg acarbose per day (40 mg TID) from the start. Usually, a dose titration period over 2 to 3 weeks is recommended to reduce the frequency and severity the GI effects when initializing treatment with acarbose.^18^ With dose titration, the reports of both flatulence and also flatulence with discharge should decrease.^18^ The GI symptoms associated with acarbose also have a clear diet component as they vary from ~3% in an Asia‐Pacific population to ~30% in a European population.^25^ The observation that there were no dropouts in the MR‐OA arms (despite the omission of dose‐titration) but two dropouts in the conventional orlistat arm could be a sign that the MR‐OA product did not aggravate conventional orlistat's side‐effects.

In this study, hunger was decreased in participants receiving MR‐OA. This is a different effect than that of conventional orally administered orlistat, which has been shown to be associated with an acutely increased appetite^14^; MR‐OA has an enteric coating that prevents gastric release of orlistat. This helps maintain a normal satiety regulation from the stomach and proximal small intestine, and a physiological fed gastric emptying rate.^14^, ^16^, ^47^, ^48^, ^49^ Although hunger was reduced, no differences in total plasma GLP‐1 concentration were found. Acarbose has been reported to enhance GLP‐1 release in healthy persons, but in type II diabetic patients, findings have varied.^50^, ^51^, ^52^, ^53^, ^54^ These studies used either higher acarbose doses (100 mg meal^−1^) or had longer treatment periods (~6 months). 50, 51, 52, 54 Other explanations for the limited effect on GLP‐1 might be the short study duration (14 days), peripheral sampling site (GLP‐1 is released in the gut), lower sensitivity to GLP‐1 secretion in persons with obesity^55^ and/or high interindividual variation. Yet another explanation for the lack of a clear effect of GLP‐1 is that the effect of MR‐OA might be more pronounced in the afternoon/evening, as indicated by the difference in hunger after lunch and dinner. Sampling was performed until about 14:00 due to constraints how much blood could be taken; therefore, any changes in GLP‐1 occurring later in the day could have been missed. Another plausible mechanism for reduced hunger is the presence of mild GI side‐effects in the MR‐OA groups, namely, nausea and gastric distension. Nausea has previously being shown to be related to appetite and weight loss.^56^ In addition, more MR‐OA participants reported mild/moderate gastric distension, and this may also have had an impact on hunger ratings.

Postprandial glucose and insulin concentrations were reduced in participants receiving MR‐OA compared with the orlistat alone, especially during day 1. This was the expected postprandial effect of the acarbose in the combination formulation. Acarbose inhibits amylase, consequently reducing the intestinal absorption rate of glucose. Both lower blood glucose concentrations as well as lower insulin concentrations have been associated with lower ratings of appetite.^57^, ^58^ Moreover, the 1 mmol difference in *C*_max_ has been shown to be equivalent of a clinically relevant change in HbA1c.^59^ Conventional orlistat has been shown to decrease the risk of developing diabetes^60^; this risk may be reduced further by combining orlistat with acarbose. It is not clear why the differences in postprandial glucose and insulin between groups were attenuated from visit 1 to visit 14; possibly, changes in meal patterns may be connected to this observation.

A short note regarding methodology: In this study, there was a focus on participants achieving a certain level of change, in addition to absolute change; analogous to weight loss studies, where not only average weight loss is of interest, but also percentage of participants who achieve either 5% or 10% weight loss.^61^ A possible bias may have been introduced because GI side‐effects were evaluated three times daily, which could have increased the participants' focus on GI side‐effects (in most published clinical trials, GI side‐effects have been assessed at longer intervals, eg, every third month).^62^, ^63^ Possibly, as a results of higher subject awareness, GI side‐effects were reported at a higher level compared with other studies.^64^ In a recent meta‐analysis,^65^ it has been shown that both acute and sustained effects on appetite aid in weight regulation. In this study, a clinically relevant change was defined as a difference in 1.5 points, which has previously been demonstrated to be related to an actual change in energy intake. 44 Furthermore, this study's 3‐d baseline evaluation of participants followed by close monitoring for 14 days is a uniquely long appetite assessment.

Some limitations with this study design need to be discussed. Firstly, at this stage of development, the trial was not fully blinded, which may have affected the subjective assessments as well as increased the “diet awareness,” especially in the conventional orlistat group. Ratings of side‐effects and hunger might have been influenced by the knowledge that the person was taking MR‐OA (which could have been perceived as the “new‐and‐improved drug”) or conventional orlistat, theoretically leading to an artificially big difference in both tolerability and hunger ratings. Limited data suggest that participants using orlistat can adjust their diet (ie, eat less fat) to avoid side‐effects, but this avoidance behaviour has only been observed for a short period (maximally a few weeks) when food intake is assessed regularly.^9^ Therefore, the improvement observed in meal patterns for participants receiving the conventional orlistat product was possibly due to awareness of conventional orlistat's mechanism of action, and this improvement was probably transient.^9^ Actual differences in tolerability as well as appetite could have been larger if no diet changes had occurred.

Secondly, the individual variation in tolerability and appetite found in the study was larger than anticipated, rendering the study somewhat underpowered. For tolerability, there was only one previous study, but with a slightly different design to base the power calculations on.^36^ A difference in number of participants achieving a clinically relevant decrease in hunger was found, although no statistical difference in hunger scores was observed. 42 So, even if the numerical difference between the MR‐OA and Xenical was in line with the number of participants achieving hunger reduction, the effect size was smaller than anticipated. This larger individual difference must be factored in for a prospective long‐term study. Lastly, only men were included in this exploratory study and the results need to be confirmed in women.

## 5 CONCLUSION

This study of OA (MR‐OA) in a MR dosage form shows that OA can be combined without aggravating their individual GI side‐effects. Moreover, MR‐OA shows promising effects regarding appetite. The effect on hunger, together with the effects on glucose and insulin levels, indicates that the specifically designed release rates of OA trigger systems involved in appetite regulation. These preliminary results need to be confirmed in a study of longer duration in a more diverse population where weight loss is the primary outcome.

## CONFLICT OF INTEREST

The authors declare the following competing financial interests: Holmbäck U, Forslund A, Grudén S, Alderborn G, Söderhäll A and Lennernäs H have equity interests in Empros Pharma AB and have acted as consultants for the company. Söderhäll A is the CEO of Empros Pharma.

## TRIAL REGISTRATION

EudraCT 2016‐001055‐50

## AUTHOR CONTRIBUTIONS

All authors took part in designing the study outline. UH, AF, GA, SG and HL designed the MR‐OA product. UH and HL wrote the manuscript. All authors have taken part in reviewing and finalizing the manuscript.

## Supporting information

Table S1. Description of the scoring in the gastric symptom score (GSS) employed in the clinical study
