# Supplementary material for: Effects of a novel combination of orlistat and acarbose on tolerability, appetite, and glucose metabolism in persons with obesity
Source: Obes Sci Pract. 2020 Feb 7;6(3):313–23. doi: 10.1002/osp4.405 (PMC7278902; doi:10.1002/osp4.405)
Supplement: Supplementary file 1 — Table S1. Description of the scoring in the gastric symptom score (GSS) employed in the clinical study [file OSP4-6-313-s001.docx]

**Supplemental table 1.**  Description of the scoring in the gastric symptom score (GSS) employed in the clinical study

| **Description of event** | **Number of daily events/average over day** | **Points** |
| --- | --- | --- |
|  |  |  |
| 1) How often have you been to the toilet for defecation? | 2 | 0 |
|  | 3 | 1 |
|  | 4 | 3 |
|  | 5 | 5 |
| 2) Have you had oily stools? | yes/no | 1/0 |
| 3) How often have you had liquid stools? | 1 | 2 |
|  | 2 | 3 |
|  | >3 | 5 |
| 4) How often have you had flatulence with discharge? | 1 | 3 |
|  | 2 | 5 |
|  | 3 | 7 |
| 5) How often have you had oily spotting? | 0 | 0 |
|  | 1 | 3 |
|  | 2 | 5 |
|  | 3 | 7 |
| 6) How often have you had fecal urgency? | 1 | 2 |
|  | 2 | 3 |
|  | 3 | 4 |
| 7) How often have you had fecal incontinence? | 1 | 3 |
|  | 2 | 5 |
|  | 3 | 7 |
| 8) Have you experienced nausea? | None | 0 |
|  | Mild | 1 |
|  | Moderate | 3 |
|  | Severe | 5 |
| 9) Have you experienced rectal pain? | None | 0 |
|  | Mild | 2 |
|  | Moderate | 3 |
|  | Severe | 5 |
| 10) Have you experienced headache? | None | 0 |
|  | Mild | 2 |
|  | Moderate | 3 |
|  | Severe | 5 |
| 11) Have you experienced gastric distention? | None | 0 |
|  | Mild | 1 |
|  | Moderate | 3 |
|  | Severe | 5 |
| 12) Have you experienced gastrointestinal pain/discomfort? | None | 0 |
|  | Mild | 2 |
|  | Moderate | 3 |
|  | Severe | 5 |
| 13) Have you experienced flatulence? | None | 0 |
|  | A little | 1 |
|  | Moderate | 3 |
|  | A lot | 5 |
